# Free Fatty Acid 4 Receptor Activation Attenuates Collagen-Induced Arthritis by Rebalancing Th1/Th17 and Treg Cells

**DOI:** 10.3390/ijms25115866

**Published:** 2024-05-28

**Authors:** Jung-Eun Lee, Ju-Hyun Lee, Jung-Min Koh, Dong-Soon Im

**Affiliations:** 1 Department of Biomedical and Pharmaceutical Sciences, Graduate School, Kyung Hee University, Seoul 02447, Republic of Korea; xkdnj1005@khu.ac.kr (J.-E.L.); ljh0620@khu.ac.kr (J.-H.L.); 2 Division of Endocrinology and Metabolism, Asan Medical Center, College of Medicine, University of Ulsan, Seoul 05505, Republic of Korea; jmkoh@amc.seoul.kr

**Keywords:** GPR120, FFA4, rheumatoid, IL-17, n-3 polyunsaturated fatty acids

## Abstract

Dietary supplementation with n-3 polyunsaturated fatty acids (PUFA) has been found to be beneficial in rodent rheumatoid arthritis models and human trials. However, the molecular targets of n-3 PUFAs and their beneficial effects on rheumatoid arthritis are under-researched. Free fatty acid receptor 4 (FFA4, also known as GPR120) is a receptor for n-3 PUFA. We aim to investigate whether FFA4 activation reduces collagen-induced rheumatoid arthritis (CIA) by using an FFA4 agonist, compound A (CpdA), in combination with DBA-1J *Ffa4* gene wild-type (WT) and *Ffa4* gene knock-out (KO) mice. CIA induced an increase in the arthritis score, foot edema, synovial hyperplasia, pannus formation, proteoglycan loss, cartilage damage, and bone erosion, whereas the administration of CpdA significantly suppressed those increases in *Ffa4* WT mice but not *Ffa4* gene KO mice. CIA increased mRNA expression levels of pro-inflammatory Th1/Th17 cytokines, whereas CpdA significantly suppressed those increases in *Ffa4* WT mice but not *Ffa4* gene KO mice. CIA induced an imbalance between Th1/Th17 and Treg cells, whereas CpdA rebalanced them in spleens from *Ffa4* WT mice but not *Ffa4* gene KO mice. In SW982 synovial cells, CpdA reduced the LPS-induced increase in pro-inflammatory cytokine levels. In summary, the present results suggest that the activation of FFA4 in immune and synovial cells could suppress the characteristics of rheumatoid arthritis and be an adjuvant therapy.

## 1. Introduction

Rheumatoid arthritis is a chronic autoimmune disease that causes progressive articular destruction [1,2]. The prevalence of rheumatoid arthritis is higher in females than males [3]. Autoreactive adaptive immune responses to citrullinated, carbamylated, and acetylated proteins and synovial inflammation lead to swelling, cartilage damage, and bone erosion [1,2,4]. In the synovial lesion, large numbers of infiltrating T cells, B cells, plasma cells, mast cells, macrophages, and synovial fibroblasts are found [5], and an imbalance between type-17-helper T cells (Th17) and Foxp3^+^ regulatory T cells has also drawn attention in the pathogenesis of rheumatoid arthritis [6,7]. Rheumatoid arthritis is considered mainly to be mediated by Th1 and Th17 cells [8,9,10]. Elucidation of articular immune responses and the involvement of a tumor necrosis factor (TNF)-α, interleukin (IL)-6, and the JAK/STAT signaling pathway that could promote synovitis have led to the development of effective biological and non-biological disease-modifying antirheumatic drugs (DMARD) [11].

On the other hand, dietary supplementations with n-3 polyunsaturated fatty acids (PUFA) have been applied in rodent rheumatoid arthritis models and human trials. In fish-oil-fed mice, the onset time of arthritis was delayed, and the incidence and severity of collagen-induced rheumatoid arthritis (CIA) was reduced compared to arthritis in corn-oil-fed mice [12]. In the DBA/1J mouse strain, which is susceptible to the development of CIA, n-3 PUFA delayed the onset of arthritis, decreased its severity, and reduced paw swelling and knee joint pathology [13]. N-3 PUFA have been shown to suppress streptococcal cell-wall arthritis in female Lew/SSN rats [14]. Endogenous conversion of n-6 into n-3 PUFA in the *fat-1* transgenic mice attenuates CIA and K/BxN serum-transfer arthritis [15,16].

In 1985, Kremer et al. stated that dietary n-3 PUFA leads to lower levels of morning stiffness and a lower number of tender joints in patients with rheumatoid arthritis [17]. Clinical benefits of n-3 PUFA are supported by a significant decrease in Disease Activity Score 28 (DAS 28 score), the number of tender joints, and a visual analogue scale score in patients with rheumatoid arthritis [18]. In studies performed in Sweden, Austria, and Norway, the intake of n-3 PUFA was associated with a good response to treatment in early and active rheumatoid arthritis patients [19,20,21]. Levels of n-3 PUFA in plasma were associated with clinical improvements in patients with rheumatoid arthritis to anti-TNF therapy by suppressing Th17 differentiation [22]. In patients with rheumatoid arthritis, the administration of n-3 PUFAs for 6 months significantly reduced levels of TNF-α, IL-1β, and prostaglandin E_2_ from the baseline and increased levels of the anti-inflammatory cytokine IL-10 [23]. Body mass index was correlated inversely with the proportions of n-3 PUFA (docosahexaenoic acid) in patients with rheumatoid arthritis [24]. The beneficial properties of n-3 PUFA in rheumatoid arthritis disease activity are also confirmed by a meta-analysis [25]. However, molecular targets of n-3 PUFAs and its beneficial effects on rheumatoid arthritis are under-researched.

Free fatty acid receptor 4 (FFA4 also known as GPR120) is a receptor for n-3 PUFA [26,27]. We have successfully proved that FFA4 is a molecular target of many beneficial effects of n-3 PUFA, including non-alcoholic hepatic steatosis, bone loss, acute lung injury, allergic asthma, atopic dermatitis, and psoriasis [28,29,30,31,32]. In the present study, we aim to investigate whether FFA4 activation could reduce CIA using an FFA4 agonist, compound A (CpdA), in combination with DBA-1J *Ffa4* gene wild-type (WT) and *Ffa4* gene knock-out (KO) mice [28,29,33].

## 2. Result

### 2.1. CpdA Activation of Ffa4 Suppressed Arthritis Development and Thickening of Foots

**Figure 1:**
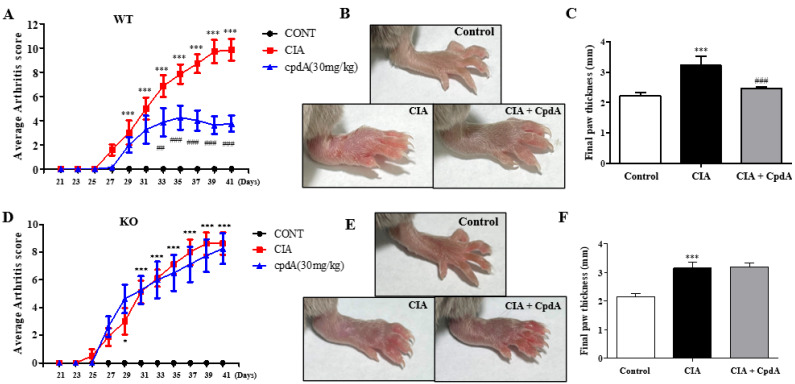
CpdA suppressed CIA-induced increases in arthritis score and paw thickness in *Ffa4* gene WT mice but not *Ffa4* gene KO mice. (**A**,**D**) Changes in arthritis score from day 21 to day 41 in *Ffa4* gene WT (**A**) and *Ffa4* gene KO (**D**) mice. (**B**,**E**) Representative pictures of *Ffa4* gene WT (**B**) and *Ffa4* gene KO (**E**) mice. (**C**,**F**) Final foot thickness on day 42 in *Ffa4* gene WT (**C**) and *Ffa4* gene KO (**F**) mice. Data were expressed as mean ± SEM (*n* = 8). *** *p* < 0.001, * *p* < 0.05 vs. the control group, ^###^ *p* < 0.001, ^##^ *p* < 0.01 vs. the CIA group.

To investigate the therapeutic potential of FFA4 in CIA, macroscopic clinical features were determined from the 21st day of the IFA injection in DBA/1J *Ffa4* gene WT mice (Figure 1A). From the 29th day, an increase in the arthritis score became significant; that is, swelling of paws. The score reached its maximum around the 40th day. The administration of CpdA showed a significant reduction in the arthritis score from the 33rd day and later compared to the CIA group, meaning there was a significant suppression of foot edema (Figure 1A). On the 42nd day, the final thickness of the paws was measured before sacrifice (Figure 1B). In the CIA group, the thickness of the paws was significantly higher than the control group, whereas the administration of CpdA significantly suppressed the increase in foot thickness induced by CIA (Figure 1B,C). In DBA/1J *Ffa4* gene KO mice, the arthritis score increased significantly and showed similar patterns to the increase in WT mice (Figure 1D); however, CpdA treatment could not suppress the increase in the arthritis score and foot thickness (Figure 1D–F), implying that CpdA suppressed the CIA-induced increase in the arthritis score and foot thickening via FFA4 activation.

### 2.2. CpdA Activation of Ffa4 Suppressed Bone Erosion, Inflammation, Cartilage Damage, and Proteoglycan Loss

**Figure 2:**
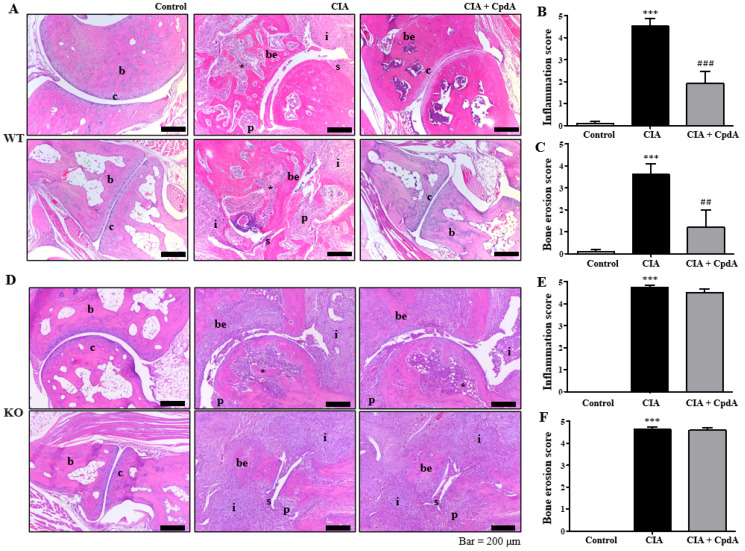
CpdA suppressed CIA-induced increase in inflammation and bone erosion score in *Ffa4* gene WT mice but not *Ffa4* gene KO mice. (**A**,**D**) H&E staining (100×) of the ankle joint in *Ffa4* gene WT (**A**) and *Ffa4* gene KO (**D**) mice. Histologic scores were evaluated in the joints based on (**B**,**E**) inflammation and (**C**,**F**) bone erosion in *Ffa4* gene WT (**B**,**C**) and *Ffa4* gene KO (**E**,**F**) mice. Data are expressed as mean ± SEM (*n* = 8). *** *p* < 0.001 vs. the control group, ^###^ *p* < 0.001, ^##^ *p* < 0.01 vs. the CIA group. b, bone; be, bone erosion; c, cartilage damage; i, inflammation; p, pannus tissue formation; s, synovial hyperplasia; *, trabecular bone loss.

Histological analysis was performed with H&E staining to determine inflammation and bone erosion. A thickening of the synovial lining called synovial hyperplasia, a representative histological characteristic of rheumatoid arthritis, was observed in the CIA group of *Ffa4* gene WT mice (Figure 2A). As synovial tissue proliferates, a lump called ‘pannus’ forms. The pannus is a histological term for synovial hypertrophy. The pannus destroys cartilage and damages the bones around the joint by including a large component of cell activity that evokes inflammation. Pannus tissue formation was observed more often in the CIA group of *Ffa4* gene WT mice (Figure 2A). However, CpdA treatment reduced the synovial hyperplasia and pannus formation in *Ffa4* gene WT mice (Figure 2A). A degree of inflammation was assessed and shown as histograms (Figure 2B). CIA significantly increased the inflammation score, whereas CpdA suppressed the increase in *Ffa4* gene WT mice (Figure 2B). Bone erosion was assessed and shown as histograms (Figure 2C). CIA significantly increased the bone erosion score, whereas CpdA suppressed the increase in *Ffa4* gene WT mice (Figure 2C). In *Ffa4* gene KO mice, the characteristics of rheumatoid arthritis of the pannus formation, bone erosion, and synovial hyperplasia increased in the CIA group; however, CpdA treatment did not suppress the characteristics (Figure 1D). That is, the inflammation and bone erosion scores were significantly increased by CIA induction, whereas CpdA treatment did not change the increase in *Ffa4* gene KO mice (Figure 1E,F). These outcomes imply that CpdA reduces CIA-induced histological changes via FFA4 activation.

**Figure 3:**
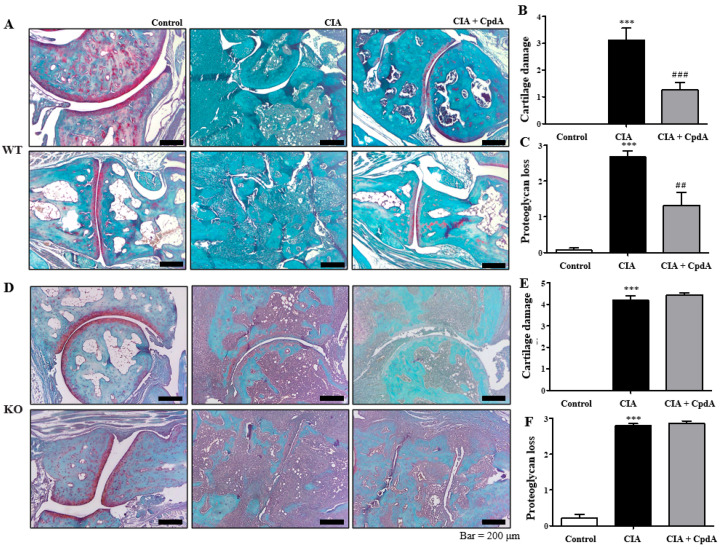
CpdA suppressed CIA-induced increase in cartilage damage and proteoglycan loss in *Ffa4* gene WT mice but not *Ffa4* gene KO mice. (**A**,**D**) Safranin-O (100×) of the ankle joint in *Ffa4* gene WT (**A**) and *Ffa4* gene KO (**D**) mice. Histologic scores were evaluated in the joints based on (**B**,**E**) cartilage damage and (**C**,**F**) proteoglycan loss in *Ffa4* gene WT (**B**,**C**) and *Ffa4* gene KO (**E**,**F**) mice. Data are expressed as mean ± SEM (*n* = 8). *** *p* < 0.001 vs. the control group, ^###^ *p* < 0.001, ^##^ *p* < 0.01 vs. the CIA group.

Furthermore, Safranin-O staining was performed to determine cartilage damage and proteoglycan loss. The levels of proteoglycan, an essential component of the extracellular matrix of articular cartilage, were stained red (Figure 3A). As shown in Figure 3A, cartilage damage was observed more often in the CIA group than in the control group, whereas CpdA treatment partly reduced the damage. Cartilage damage was assessed, and histograms are shown in Figure 3B. CIA significantly increased cartilage damage, whereas CpdA suppressed the damage increase in *Ffa4* gene WT mice (Figure 3B). Compared to the control group, proteoglycan disappeared clearly in the CIA group, whereas CpdA administration partly protected against the loss of proteoglycans in *Ffa4* gene WT mice (Figure 3A,C). In *Ffa4* gene KO mice, CIA induced cartilage damage and proteoglycan loss similar to the WT mice; however, CpdA administration did not protect against cartilage damage or the loss of proteoglycan (Figure 3D–F). Again, the results imply that CpdA protects against CIA-induced cartilage damage and proteoglycan loss via FFA4 activation.

### 2.3. CpdA Activation of Ffa4 Suppressed Enlargement of Spleens and Rebalanced Th1/Th17 and Treg Cells in Spleens

**Figure 4:**
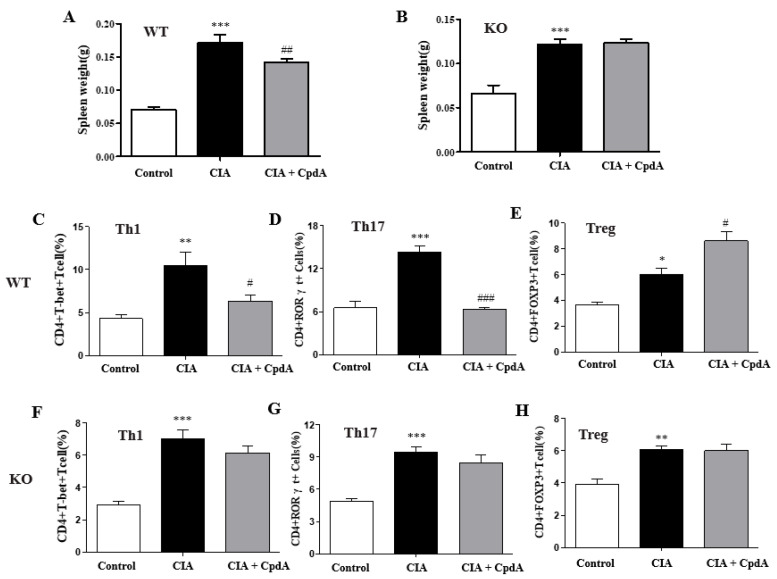
CpdA suppressed CIA-induced spleen enlargement and regulated the imbalance of Th1/Th17 and Treg cells in *Ffa4* gene WT mice but not *Ffa4* gene KO mice. (**A**,**B**) Spleen weights of *Ffa4* gene WT (**A**) and *Ffa4* gene KO (**B**) mice. (**C**,**F**) Percentage of CD4^+^T-bet^+^ Th1 cells in *Ffa4* gene WT (**C**) and *Ffa4* gene KO (**F**) mice. (**D**,**G**) Percentage of CD4^+^T RORγt^+^ Th17 cells in *Ffa4* gene WT (**D**) and *Ffa4* gene KO (**G**) mice. (**E**,**H**) Percentage of CD4^+^T FoxP3^+^Treg cells in *Ffa4* gene WT (**E**) and *Ffa4* gene KO (**H**) mice. The results are presented as the mean ± SEM (*n* = 8). *** *p* < 0.001, ** *p* < 0.01, * *p* < 0.05 vs. the control group, ^###^ *p* < 0.001, ^##^ *p* < 0.01, ^#^ *p* < 0.05 vs. the CIA group.

Next, we measured the spleen, an important organ in the immune system. Compared to the control group, the weight of the spleens significantly increased in the CIA group of *Ffa4* gene WT and *Ffa4* gene KO mice (Figure 4A,B). The administration of CpdA significantly reduced the increase in the spleen weight in *Ffa4* gene WT mice but not *Ffa4* gene KO mice (Figure 4A,B). In addition, the population of Th1, Th17, and Treg cells was determined in the spleen by FACS analysis because the imbalance between different T cells, like type 1 and 17 T-helper cells (Th1 and Th17) versus regulatory T cells (Treg), is important for rheumatoid arthritis progression and development [34]. By CIA induction, the population of CD4^+^T-bet^+^ Th1 cells, CD4^+^RORγt^+^ Th17 cells, and CD4^+^FoxP3^+^ Treg cells significantly increased, whereas CpdA reduced the increase in Th1 and Th17 cells but augmented the increase in Treg cells in *Ffa4* gene WT mice (Figure 4C–E). In *Ffa4* gene KO mice, CIA induction increased the population of Th1, Th17, and Treg cells significantly, whereas CpdA did not reduce the increase in Th1/Th17 cells and did not augment the increase in Treg cells (Figure 4F–H). An endogenous increase in Treg cells by CIA induction in the spleens may result from compensatory mechanisms balancing the Th17/Treg ratio. A CIA-induced augmentation in the increase in Treg cells was only observed in *Ffa4* gene WT mice but not *Ffa4* gene KO mice, implying the involvement of FFA4.

### 2.4. CpdA Activation of Ffa4 Suppressed mRNA Expression Levels of Pro-Inflammatory Cytokines in Foot Tissues

**Figure 5:**
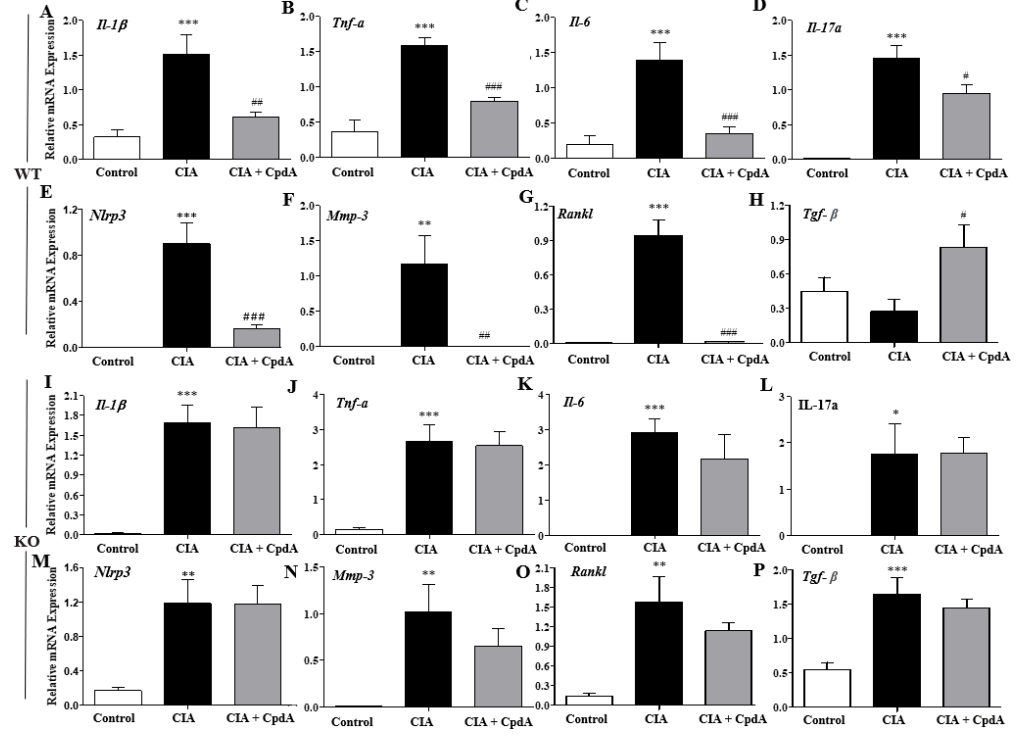
CpdA suppressed CIA-induced increase in inflammatory cytokine levels in *Ffa4* gene WT mice but not *Ffa4* gene KO mice. The mRNA levels of cytokines were quantified as ratios to the *Gapdh* mRNA level. (**A**,**I**) *Il-1β*, (**B**,**J**) *Tnf-a*, (**C**,**K**) *Il-6*, (**D**,**L**) *Il-17a*, (**E**,**M**) *Nlrp3*, (**F**,**N**) *Mmp-3*, (**G**,**O**) *Rankl*, and (**H**,**P**) *Tnf-β* in *Ffa4* gene WT (**A**,**H**) and *Ffa4* gene KO (**I**,**P**) mice. The level of significance was set at *** *p* < 0.001, ** *p* < 0.01, * *p* < 0.05 vs. the control group, ^###^ *p* < 0.001, ^##^ *p* < 0.01, ^#^ *p* < 0.05 vs. the CIA group.

As rheumatoid arthritis is a systemic autoimmune disease, and changes in Th1, Th17, and Treg cell populations in the spleens may result in changes in pro-inflammatory cytokine levels in feet, mRNA expression levels of pro-inflammatory cytokines were determined in the foot tissues obtained on the 42nd day. The mRNA levels of pro-inflammatory Th1 (*Il-1β*, *Tnf-α*, and *Il-6*) and Th17 (*Il-17a*) cytokines were increased in the CIA group when compared with the control group, whereas CpdA administration suppressed the increased cytokine levels in *Ffa4* gene WT mice (Figure 5A,D). In addition, the mRNA levels of *Nlrp3*, an inflammasome-related protein, *Mmp-3*, a protease that executes joint destruction, and *Rankl*, a cytokine that stimulates osteoclasts to carry out the bone resorption process, were increased in the CIA group, whereas CpdA significantly suppressed these increases in *Ffa4* gene WT mice (Figure 5E,G). The levels of *Tgf-β*, an anti-inflammatory cytokine from Treg cells, decreased slightly but not significantly by CIA induction, whereas CpdA administration significantly increased the expression of *Tgf-β* compared to the CIA group in *Ffa4* gene WT mice (Figure 5H). In *Ffa4* gene KO mice, CIA induced an increase in Th1/Th17 cytokine levels along with *Nlrp3*, *Mmp-3*, and *Rankl*, similarly to WT mice; however, CpdA administration did not reduce the increase (Figure 5,I,O). The mRNA levels of *Tgf-β* increased significantly in the CIA group compared to the control group of *Ffa4* gene KO mice (Figure 5P), which is contrasting to the non-significant change in the CIA group compared to the control group of *Ffa4* gene WT mice. CpdA administration did not change the increase in *Tgf-β* mRNA levels compared to the CIA group in *Ffa4* gene KO mice (Figure 5P).

### 2.5. CpdA Activation of Ffa4 Suppressed Serum IgG Levels

**Figure 6:**
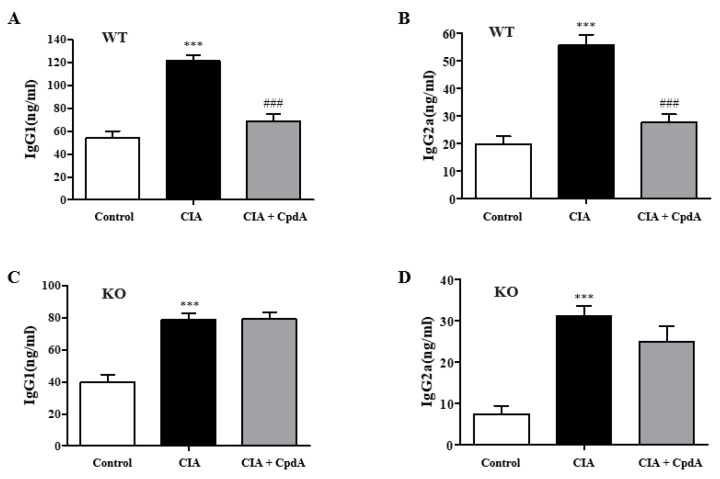
CpdA suppressed CIA-induced increase in IgG1 and IgG2a levels in the serum of *Ffa4* gene WT mice but not *Ffa4* gene KO mice. The blood was collected on day 42. Serum levels of IgG1 (**A**,**C**) and IgG2a (**B**,**D**) were measured by ELISA in *Ffa4* gene WT (**A**,**B**) and *Ffa4* gene KO (**C**,**D**) mice. Data were expressed as mean ± SEM (*n* = 8). *** *p* < 0.001 vs. the control group, ^###^ *p* < 0.001 vs. the CIA group.

Rheumatoid arthritis is an immune response derived from autoantibodies, which incorrectly target and react to antigens in the joints. IgG, the most common autoantibody, is frequently found in high levels of rheumatoid arthritis. Different IgG subclasses play roles in complementing immune and phagocytosis functions and promoting inflammation. The levels of IgG1 and IgG2a subclasses, which are important in complement activation, were determined. Both IgG1 and IgG2a levels were significantly increased in the CIA group compared to the control group, whereas CpdA significantly reduced the levels in *Ffa4* gene WT mice (Figure 6A,B). In *Ffa4* gene KO mice, both IgG levels were increased by CIA induction; however, CpdA treatment could not reverse the increase (Figure 6C,D).

### 2.6. CpdA Activation of Ffa4 Suppressed mRNA Expression Levels of Inflammatory Cytokines in SW982 Human Synovial Cells

**Figure 7:**
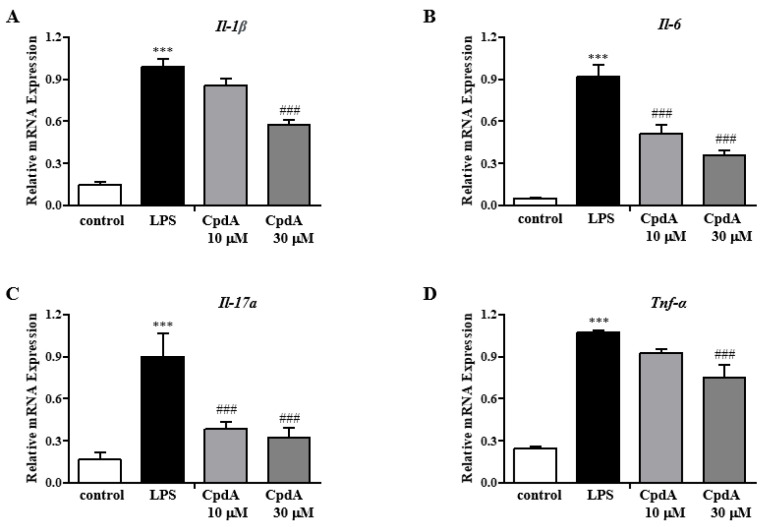
CpdA inhibited LPS-induced increase in pro-inflammatory cytokines mRNA expression in SW982 Cells. SW982 cells were seeded at 1 × 10^5^/mL. After 24 h, CpdA (30 mM) with or without AH7614 (10 mM) was added, and then, LPS (100 ng/mL) was added and incubated for 30 min. qPCR was used to confirm mRNA expression levels of inflammatory cytokines in SW982 cells. (**A**) *IL-1β*, (**B**) *IL-6*, (**C**) *IL-17A*, and (**D**) *TNF-α*. The results are presented as the mean ± SEM (*n* = 5). *** *p* < 0.001 vs. the Control group; ^###^ *p* < 0.001 vs. the LPS induced group.

A hyperplastic synovial membrane, a main characteristic of rheumatoid arthritis, secretes cytokines that facilitate cartilage damage and most likely mediate the chronicity of the disease. To check whether CpdA could regulate inflammatory responses in the synovial membrane, a human synovial cell line SW982 was utilized. Inflammatory cytokine mRNA levels were determined in LPS-stimulated SW982 cells by qRT-PCR. LPS treatment increased the expression levels of various inflammatory cytokines (*IL-1β*, *TNF-α*, *IL-6*, and *IL-17a*), whereas CpdA significantly suppressed these (Figure 7A–D). In the presence of AH7614, a selective antagonist of FFA4, CpdA-induced suppression was blunted (Figure 7A–D). In SW982 cells, cell viability was not affected by CpdA at the concentrations used.

## 3. Discussion

N-3 PUFAs could alleviate the severity of disease in rodent rheumatoid arthritis models [12,13,14,15,16,35] and human trials [17,18,19,20,21]. In the present study, CIA-induced characteristic changes in rheumatoid arthritis were observed in DBA-1J *Ffa4* gene WT and *Ffa4* gene KO mice. The administration of CpdA, a selective agonist of FFA4, significantly suppressed the changes; that is, it changed the arthritis score, foot edema, synovial hyperplasia, pannus formation, proteoglycan loss, cartilage damage, and bone erosion in *Ffa4* gene WT mice but not *Ffa4* gene KO mice, suggesting that the CpdA activation of FFA4 could alleviate the severity of rheumatoid arthritis. Macroscopic and microscopic changes by CIA and their suppression by CpdA were mechanistically supported by the changes in immune responses, such as pro-inflammatory cytokines and a balance of Th1/17 and Treg cells.

In patients with rheumatoid arthritis, dietary supplementation with n-3 PUFA results in significantly decreased IL-1β levels in plasma [36], and in healthy volunteers, diets enriched in fish oil n-3 PUFA lead to reduced TNF-α and IL-1β levels [37]. In previous studies, pro-inflammatory cytokine productions, including Il-17, Tnf-α, Ifn-γ, Il-1β, Il-2, Il-6, Il-23, and Il-27 from immune cells such as T cells, macrophages, dendritic cells, and neutrophils have been shown to be suppressed by n-3 PUFAs [38]. The involvement of FFA4 in the CpdA-induced suppression of pro-inflammatory cytokines has been shown in allergic asthma (*Il-4*, *Il-5*, *Il-13*, *Ifn-g*, and *Il-17a*), atopic dermatitis (*Il-4*, *Il-13*, *Ifn-g*, and *Il-17a*), and psoriasis (*Il-17a*, *Il-22*, *Il-23*, *Il-1b*, *Ifn-g*, and *Tnf-a*) models using *Ffa4* gene KO mice [30,39]. In the present study, levels of the representative pro-inflammatory Th1/Th17 cytokines (*Il-1b*, *Tnf-a*, *Il-6*, and *Il-17a*) in a CIA arthritis model were increased by CIA and reversed by CpdA in an *Ffa4* gene-dependent manner. In addition, mRNA levels of *Nlrp3* (an inflammasome component), which are necessary for the sustained production of IL-1b by the inflammasome, were similarly changed by CIA and CpdA. This result is supported by previous reports that suggest n-3 PUFAs reduces Nlrp3 activation by FFA4 and β-arrestin-2 in high-fat-diet-induced type 2 diabetes, acute cerebral infarction, and epileptic animal models [40,41]. CIA increased mRNA levels of *Mmp3*, a cartilage-degrading proteinase, and CpdA reversed the increase in an *Ffa4* gene-dependent manner, which supports the previous observation that EPA reduces the mRNA levels of *Mmp3* in bovine chondrocytes [42]. In the present CIA model, mRNA levels of *Rankl*, a key factor for osteoclast differentiation, were increased by CIA and reserved by CpdA in an *Ffa4* gene-dependent manner, which correlated with the observation of EPA inhibition of Rankl expression in osteoblasts in an inflammatory environment [43]. Changes in *Tgf-β* expression, an anti-inflammatory and tissue-resolving factor, by CIA and CpdA are intriguing between *Ffa4* gene WT and *Ffa4* gene KO mice. In *Ffa4* gene WT mice, mRNA levels of *Tgf-β* were slightly, but not significantly, decreased by CIA; however, CpdA increased its expression in an *Ffa4*-dependent manner, which supports the macroscopic and microscopic changes in arthritis. However, in *Ffa4* gene KO mice, mRNA levels of *Tgf-β* were increased significantly by CIA, and there was no change by CpdA treatment. Thus, mRNA levels of *Tgf-β* were increased by CIA in *Ffa4* gene KO mice but not in *Ffa4* gene WT mice. Why and how an *Ffa4* gene deficiency increases the *Tgf-β* mRNA expression in inflammatory conditions are questions worth investigating further. In summary, decreased expression in pro-inflammatory and cartilage-destroying cytokines (*Il-1β*, *Tnf-α*, *Il-6*, *Il-17a*, *Nlrp3*, *Mmp3*, and *Rankl*) and the increased expression of an anti-inflammatory *Tgf-β* by CpdA support the Ffa4-dependent amelioration of inflammatory arthritis responses.

Such changes in cytokine levels may be a result of balancing Th1/Th17 and Treg cells. In previous studies, n-3 PUFAs decreased T-cell proliferation/activation and differentiation into Th1/Th17 phenotypes and increased Treg numbers [38]. In vitro studies showed that n-3 PUFAs can inhibit T-cell activation and proliferation [35] and suppress Th1-cell development [44,45]. Docosahexaenoic acid (DHA, an n-3 PUFA) supplementation decreases the proportion of Th17 cells and enhances the proportion of Treg cells in the spleen [46]. *Ffa4* gene deficiency promotes Th17-cell differentiation and attenuates the population of Treg cells [46]. N-3 PUFAs reduce the population of Th1/Th17 cells and increase the population of Treg cells in recipient mice of heart transplantation [47]. Dietary n-3 PUFAs also decrease Th17-cell accumulation during chronic experimental colitis [48,49]. Endogenous conversion of n-6 to n-3 PUFA in *fat-1* transgenic mice restores the balance between Th17 and Treg in CIA [16]. Previously, we have demonstrated that CpdA activation of Ffa4 suppressed allergic asthma, which is driven by Th2 cytokines, and psoriasis, which is driven by Th17 cells [30,39]. In addition, CpdA treatment suppressed the differentiation of naïve T cells into Th17 cells in vitro and increased the Th17-cell population in a psoriasis-like animal model in an *Ffa4* gene-dependent manner [39]. Also, CpdA treatment increased the population of Treg cells in an atopic-dermatitis-like animal model in an *Ffa4* gene-dependent manner [29]. Therefore, CIA-induced an imbalance between Th1/17 and Treg cells, and their rebalancing by CpdA is considered to play a key role in the immune responses of cytokine production and subsequent histological changes in CIA mice. The FFA4-mediated rebalancing of Th1/Th17 and Treg cells might be a common way to suppress inflammatory immune responses by n-3 PUFAs and CpdA.

Changes in inflammatory Th1/Th17-cell populations and anti-inflammatory Treg-cell populations could be a result of not only T-cell differentiation but also the suppression of dendritic cell activation by n-3 PUFA [50]. DHA treatment induces a tolerogenic dendritic cell phenotype in vivo through FFA4 and correspondingly promotes the induction of Treg cells through an increased expression of TGF-β [46]. DHA treatment suppresses the expression of MHCII, CD80, CD86, and CD40 and induces the expression of CD45RB and PD-L2, indicating regulatory dendritic cells [51]. Differentiation of naïve T cells into Th1 and Th17 cells was significantly suppressed in co-culture with DHA-treated dendritic cells [52]. On the other hand, DHA-treated dendritic cells increased Foxp3 expression and the proportion of Foxp3^+^ Treg cells [53,54]. Therefore, the present results might partly be a result of tolerogenic dendritic cells through Ffa4.

The formation of a hyperplastic synovial membrane is also an important tissue response in rheumatoid arthritis because it facilitates the development of structural damage and mediates the chronicity of the disease by producing cytokines. In human SW982 synovial cells, inflammatory responses by LPS were suppressed by CpdA in an FFA4-dependent manner. Therefore, the direct activation of FFA4 in synovial cells could also contribute to the suppression of synovial hyperplasia and the subsequent attenuation of rheumatoid arthritis in the CIA model.

In a study by Wannick et al., CpdA did not affect disease development in K/BxN serum transfer arthritis mice [55], which is in contrast to the efficacy of CpdA on CIA in the present study. There are two different factors between the study of Wannick et al. and our study [55]. One is mouse strains (DBA-1J and C57BL/6), and the other is arthritis models (type II collagen vs. K/BxN serum). However, these could not explain the contradictory results. In a similar case, we previously showed the effectiveness of CpdA on psoriasis-like dermatitis in an Ffa4-dependent manner [39]; however, Wannick et al. could not provide positive results using *Ffa4* gene KO mice in the same psoriasis-like dermatitis and an antibody transfer pemphigoid disease-like dermatitis [55]. In an osteoarthritis model induced by anterior cruciate ligament transection surgery, *Ffa4* gene KO mice showed accelerated development of osteoarthritis; that is, cartilage degeneration, inflammation, and subchondral bone aberrant changes [56]. DHA FFA4 activation also exhibited anti-inflammatory effects in primary human chondrocytes in vitro [56]. GW9508 and TUG891, other FFA4 agonists, rescued the expression of aggrecan and type II collagen by preventing the reduction in SOX9 expression, implying a potential therapeutic option to prevent cartilage degradation [57]. In combination with the present results and other publications, Ffa4 activation would be a therapeutic option to modify rheumatoid arthritis development.

## 4. Materials and Methods

### 4.1. Materials

CpdA (3-[2-chloro-5-(trifluoromethoxy)phenyl]-3-azaspiro [5.5]undecane-9-acetic acid) was purchased from Cayman Chemical (Ann Arbor, MI, USA). Bovine type II collagen, complete Freunds adjuvant (CFA), and incomplete Freunds adjuvant (IFA) were obtained from Chondrex Inc. (Woodinville, WA, USA). Other chemicals were purchased from Sigma-Aldrich (St. Louis, MO, USA).

### 4.2. Cells

Human synovial SW982 cells were obtained from the American Type Culture Collection (ATCC, Manassas, VA, USA). The cells were cultured and maintained in DMEM with a 10% heat-inactivated fetal bovine serum and 1% penicillin–streptomycin in a humidified atmosphere with 5% CO_2_ at 37 °C.

### 4.3. Mouse Strain

FFA4 knock-out mice (TF0224) were purchased from Lexicon Pharmaceuticals (Woodlands, TX, USA) and backcrossed to DBA/1J mice for eight generations [28,29]. All animals were housed in a laboratory animal facility at Kyung Hee University and provided with food and water ad libitum. The mice were housed two per cage in standard plastic cages with sawdust as bedding, with the environment maintained under controlled conditions of temperature ranging between 22–24 °C, a humidity level of 60 ± 5%, and alternating light/dark cycles (lights were on between 7:00 h and 19:00 h). The mice were also provided with standard laboratory chow and water. The animal protocol was reviewed by the Institutional Animal Care Committee of Kyung Hee University with respect to the ethics of the procedures used and care (KHSASP-23-437).

### 4.4. Treatment of Human Synovial SW982 Cells

SW982 cells were seeded at 1 × 10^5^ cells in a 6-well plate and cultured overnight. Then, the cells were washed with PBS and treated with LPS (100 ng/mL) overnight. CpdA was treated 30 min before LPS treatment.

### 4.5. Induction of Rheumatoid Arthritis in DBA/1J Mice and CpdA Administration

CIA is a common autoimmune animal model used to study rheumatoid arthritis. Male DBA/1J mice weighing 23 to 25 g in age from 7 to 9 weeks were randomly divided into three experimental groups (*n* = 8 per group): a control group, a CIA group, and a CpdA (30 mg/kg) + CIA group. CFA and bovine type II collagen were mixed in the same ratio, and an emulsion was prepared as described previously [58]. Mice were anesthetized with Avertin and injected with 100 mL of emulsion into the tail, approximately 1.5 cm distal from the base of the tail. The second injection was conducted with IFA on the 21st day at 2 cm from the base of the tail until the tip reached 1.5 cm from the base. CpdA administration was carried out 30 min before the emulsion injection. CpdA treatment began on the 21st day and continued until the 41st day. Clinical scores and body weights were evaluated from the 21st day until the 41st day.

### 4.6. Measurement of the Severity of Arthritis

After the booster shot on day 21, mice were scored for arthritis severity every other day. Arthritis scores are determined on the extremities of each mouse, and the score for each mouse is the sum of limb scores (range, 0–16 points, with a maximum score in each mouse of 16) [59,60]. Arthritis scores were assigned as follows: 0 = no erythema or swelling, 1 = erythema and mild swelling confined to the tarsals, ankle, or paw joint, with mild swelling at the single limb, 2 = erythema and mild swelling extending from the ankle to the tarsals, or erythema and mild swelling of more than one toe, 3 = erythema and moderate swelling extending from the ankle to the metatarsal joints or the whole paw with swelling and obvious erythema, and 4 = erythema and the whole paw with severe swelling encompassing the ankle, foot, and digits, or ankylosis of the limb, and dysfunction of the above joints [59,60].

### 4.7. Histological Assessment of Arthritis

The feet of DBA/1J mice were submitted to the Korean non-clinical technology solution center corporation (Seoul, Republic of Korea) for paraffin sectioning. Paraffin-sectioned foot slides were used to confirm histological changes in rheumatoid arthritis through Safranin-O staining. Safranin-O staining was performed according to the protocol of the NovaUltra Safranin-O Stain Kit from IHC WORLD (cat. IW-3011, Ellicott City, MD, USA). H&E staining was performed on another paraffin-sectioned foot slide. For deparaffinization, the sections were placed in xylene for five minutes and dehydrated with EtOH. After treatment with a hematoxylin solution, the sections were washed with tap water and re-dehydrated with EtOH. Then, after being stained with Eosin Y, the sections were fixed with EtOH and encapsulated with Permount. The scores (0 to 5) of inflammation, pannus, cartilage, and bone erosion were measured according to the indicated index of reference [61].

Inflammation and bone erosion were scored on a scale of 0 to 5; 0 = normal, 1 = minimal (infiltration of local inflammatory cells or mild swelling), 2 = mild (infiltration of local inflammatory cells and mild swelling), 3 = moderate (obvious bone resorption of trabecular and cortical bone, without defects in cortex or loss of trabeculae), 4 = marked (significant inflammatory cell infiltration and damage to the cortical bone and trabecular bone), and 5 = severe (severe infiltration of inflammatory cells and destruction of the entire skeleton) [62,63,64]. Cartilage damage was calculated by the loss of Safranin-O staining that was scored on a semi-quantitative scale from 0 to 4; 0 = intact, 1 = minor, 2 = moderate, 3 = high, and 4 = severe [65,66].

### 4.8. Flowcytometric Analysis

To determine the T-cell population, single cells isolated from spleens were stained with an FITC-labeled rat antibody against CD4 (cat. 11-0041-82, eBioscience, San Diego, CA, USA) at 4 °C for 20 min. The cells were fixed at room temperature for 1 h using an intracellular fixation buffer (cat. 00-8222-49, eBioscience). After fixation, the cells were permeabilized using a permeabilization buffer (cat. 88-8824-00, eBioscience) and stained at room temperature for 1 h with APC-labeled rat anti-Foxp3 (cat. 17-5773-82, eBioscience), eFluor 450-labeled rat anti-T-bet (cat. 48-5825-82, eBioscience), or APC-labeled rat anti-RORγt (cat. 17-6988-82). The cells were analyzed using a CytoFLEX Flow cytometer (Beckman Coulter, Brea, CA, USA).

### 4.9. Quantitative Real-Time PCR

To assess the expression of inflammatory markers in the feet of mice by qRT-PCR, a first-strand cDNA was synthesized from total RNA isolated using a TRIzol reagent (Invitrogen, Waltham, MA, USA); total RNA was isolated from the feet or SW982 human synovial cells. The RNA was transcribed reversely into cDNA using MMLV reverse transcriptase (Promega, Madison, WI, USA). Thunderbird Next SYBR qPCR Mix (Toyobo, Osaka, Japan) was used for qRT-PCR in a CFX Connect Real-Time System (Bio-Rad, Hercules, CA, USA). Specific primers of *Mus musculus Il-1β* (sense 5′- CAG GCA GGC AGT ATC ACT CA -3′, antisense 5′- TGT CCT CAT CCT GGA AGG TC -3′), *Tnf-α* (sense 5′- ACG GCA TGG ATC TCA AAG AC -3′ antisense 5′- GTG GGT GAG GAG CAC GTA GT -3′), *Il-6* (sense 5′- CTG ATG CTG GTG ACA ACC AC -3′, antisense 5′- TCC ACG ATT ACC CAG AGA AC -3′), *Il-17a* (sense 5′- CAGCGTGTCCAAACACTGAG -3′ antisense 5′- CGGTTGAGGTAGTCTGAGGG -3′), *Tgf-β* (sense 5′- GGA CTC TCC ACC TGC AAG AC -3′, antisense 5′- GAC TGG CGA GCC TTA GTT TG -3′), *Mmp-3* (sense sense 5′- CAG GTG TGG TGT TCC TGA TG -3′, antisense 5′- TTT CAA TGG CAG AAT CCA CA -3′), *Rankl* (sense 5′- GCA GAA GGA ACT GCA ACA CA -3′, antisense 5′- GAT GGT GAG GTG TGC AAA TG -3′), *Nlrp-3* (sense 5′- ATG CTG CTT CGA CAT CTC CT -3′, antisense 5′- GTT TCT GGA GGT TGC AGA GC -3′), and *Gapdh* (sense 5′-AAC TTT GGC ATT GTG GAA GG-3′, antisense 5′-GGA TGC AGG GAT GAT GTT CT-3′), *Homo sapiens GAPDH* (sense 5′- CCA CCC AGA AGA CTG TGG AT -3′ antisense 5′- TTC AGC TCA GGG ATG ACC TT -3′), *IL-1β* (sense 5′- GGA CAA GCT GAG GAA GAT GC -3′ antisense 5′- TCG TTA TCC CAT GTG TCG AA -3′), *IL-6* (sense 5′- GAA AGC AGC AAA GAG GCA CT -3′ antisense 5′- TTT CAC CAG GCA AGT CTC CT -3′), *IL-17A* (sense 5′- ATG AAC TCT GTC CCC ATC CA -3′ antisense 5′- CCC ACG GAC ACC AGT ATC TT -3′), and *TNF-α* (sense 5′- AAC CTC CTC TCT GCC ATC AA -3′ antisense 5′- GGA AGA CCC CTC CCA GAT AG -3′) were used. Thermal-cycling conditions were as follows: one cycle at 95 °C for 4 min, 40 cycles at 95 °C for 30 s and at 57 °C for 30 s, and one cycle at 95 °C for 30 s. The 2^−ΔΔCt^ method was used to calculate each gene expression.

### 4.10. Enzyme-Linked Immunosorbent Assay (ELISA)

Serum was stored at –80 °C until use. Mouse serum IgG1 and IgG2a levels were determined using ELISA kits (eBioscience, San Diego, CA, USA, IgG1: cat. no. 88-50410-88, IgG2a: cat. no. 88-50420-88). Avidin–horseradish peroxidase was used, and absorbance was measured at 450 nm.

### 4.11. Normality and Statistical Analysis

The results were expressed as the mean ± standard error of the mean (SEM) of eight measurements for the animal experiments. The Kolmogorov–Smirnov (KS) test was performed to investigate whether the data passed the normality test. The statistical significance of the differences was determined by a two-way ANOVA analysis and Tukey’s multiple comparison test (GraphPad Software version 9.0, La Jolla, CA, USA). Statistical significance was set at *p* values < 0.05. * indicates a significant difference compared to the vehicle-treated group; # indicates a significant difference compared to the CIA group.

## Data Availability

The original contributions presented in the study are included in the article, further inquiries can be directed to the corresponding author.
